# A Brain-Inspired Model of Hippocampal Spatial Cognition Based on a Memory-Replay Mechanism

**DOI:** 10.3390/brainsci12091176

**Published:** 2022-09-01

**Authors:** Runyu Xu, Xiaogang Ruan, Jing Huang

**Affiliations:** 1 Faculty of Information Technology, Beijing University of Technology, Beijing 100124, China; 2 Beijing Key Laboratory of Computational Intelligence and Intelligent System, Beijing 100124, China

**Keywords:** brain-inspired, hippocampus, place cell, memory replay, spatial cognition, autonomous navigation

## Abstract

Since the hippocampus plays an important role in memory and spatial cognition, the study of spatial computation models inspired by the hippocampus has attracted much attention. This study relies mainly on reward signals for learning environments and planning paths. As reward signals in a complex or large-scale environment attenuate sharply, the spatial cognition and path planning performance of such models will decrease clearly as a result. Aiming to solve this problem, we present a brain-inspired mechanism, a Memory-Replay Mechanism, that is inspired by the reactivation function of place cells in the hippocampus. We classify the path memory according to the reward information and find the overlapping place cells in different categories of path memory to segment and reconstruct the memory to form a “virtual path”, replaying the memory by associating the reward information. We conducted a series of navigation experiments in a simple environment called a Morris water maze (MWM) and in a complex environment, and we compared our model with a reinforcement learning model and other brain-inspired models. The experimental results show that under the same conditions, our model has a higher rate of environmental exploration and more stable signal transmission, and the average reward obtained under stable conditions was 14.12% higher than RL with random-experience replay. Our model also shows good performance in complex maze environments where signals are easily attenuated. Moreover, the performance of our model at bifurcations is consistent with neurophysiological studies.

## 1. Introduction

Research on autonomous robot navigation has received much attention. Spatial cognition and navigation, being among the most basic abilities of animals, have received extensive attention from researchers. Bionic navigation strategies and brain-like spatial cognitive models have become popular topics in recent years [1].

Hippocampus position cells are an important part of mammalian allogenic central representation encoding local space that can generate specific discharges to establish mapping relationships with specific areas in physical space, thus forming cognitive maps, the bases of animal cognitive space and navigation. The formation of cognitive maps in the hippocampus is considered to be the neurophysiological basis of cognitive mapping [2]. One widely accepted theory of hippocampus function holds that location cell representations are formed during an animal’s first exposure to a new environment and are subsequently replayed offline to support the consolidation of memories and future behavior [3].

Some researchers believe that the core region of goal-directed navigation is not in the hippocampus but is more dependent on the prefrontal cortex, which receives positioning information from the hippocampus, evaluates and sparsely codes the cognitive map generated by the hippocampus, and thus generates navigation planning for specific targets. Burnod et al. first proposed a cortical architecture model called the “cortical automata” that shows the “call tree” process of path planning [4]. Subsequently, Michael et al. proposed a functional model of the prefrontal cortex that uses cognitive maps as input to guide agent behavior [5]. Martinet et al. proposed a neuronal architecture based on back propagation and forward search that provides a functional framework for explaining the activity of the prefrontal cortex and hippocampus during navigation [6]. These studies focused on the role of the prefrontal cortex in path planning, its thinning of the encoding of complex information in the hippocampus and its dependence on attenuated signals to transmit information in the environment.

However, these studies have overlooked the reactivation function of the mammalian hippocampus itself; these models combine the functions of the prefrontal cortex but suffer from signal attenuation. Additionally, long-distance signaling makes reward information more susceptible to neuronal noise, which in turn speeds up signal attenuation. This signal attenuation is a fatal problem in these models, especially in complex environments. Once the signal decreases to zero, it marks the failure of the navigational mission.

The problem of signal attenuation not only affected the above studies but also is a common problem in navigation research. Navigation models including reinforcement learning architecture [7] also have the problem of signal attenuation.

The signal attenuation problem is similar to how we switch from short-term memory to long-term memory: If previously generated short-term memories are not repeatedly stimulated over a long period of time, they are quickly forgotten. Because place cell activity shows an increase in temporal structure after task execution, hippocampus activity during replay is thought to be involved in memory consolidation [8,9,10,11,12,13]. Later work led to neural activity: place cells fire in an orderly fashion—during sleep. Arranging cell discharges in the order that the rats encountered them during the task is known as routing replay [8,9]. Hippocampus replay has been examined in simple runway tasks [8,9,10,11].

Studies have shown that place cells are activated not only during active navigation but also during passive states including sleep [8,11], and when awake but not moving [14], place cells are also activated. This reactivation function is clearly correlated with mammalian learning rates and is compatible with the consolidation theory of long-term memory [15]. In addition, Wood et al. and Frank et al. found another anticipatory encoding mechanism in the hippocampus [16,17]. The reactivation of the hippocampus is usually associated with the mechanism of planning future behavior. Moreover, during passive-state reactivation, the stimulus and the reward system are associated with position preference [18].

It has been found that rats with damaged prefrontal cortex can still complete basic Morris water maze tests [19]. Therefore, in order to facilitate the modeling of the hippocampus, the influence of the prefrontal cortex on navigation path planning was not taken into account in this study, and we focused on the hippocampus memory replay.

Inspired by the remapping of hippocampus place cells, this paper proposed a mechanism of memory replay and established a hippocampus–striatum model from the perspective of neurophysiology to reduce the impact of signal attenuation on navigation. In this model, hippocampus place cell action sequences were used as tracks, and memory sequences were formed and stored in the memory vault. The memory sequences in the memory vault were integrated and reconstructed to form virtual track sequences to simulate the ability of animals to predict future spatial states through past experience. According to the correlation between the sequence and the reward information, the real memory sequence and the virtual track sequence were reactivated in the hippocampus to enhance the transmission of the reward information in the cognitive map. We call this algorithm the Memory-Replay Mechanism.

The main contributions of this paper are as follows:

- We establish a new hippocampus–striatum spatial cognitive navigation model based on reward learning. The model is brain-inspired and can increase the strength of signal transmission in navigation and reduce the impact of signal attenuation on environmental cognition.
- In this model, we propose the Memory-Replay Mechanism, which is inspired by the reactivation function of place cells in the hippocampus. This mechanism can consolidate the memory for the agent and automatically generate a “virtual path” according to the reward information.
- The spatial cognition and navigation ability of our model were proved with the experiments. Compared with the conventional reinforcement learning methods and other brain-inspired methods, our model has apparent advantages in path planning.
- Our model provides a possible explanation for how animal brains work in goal-directed navigation tasks from the computation perspective, as the signal-propagation patterns of our model in complex mazes are consistent with the phenomena of place cell reactivation.

The remainder of this article is organized as follows.

The Section 1 of the paper is an introduction, the Section 2 is related works and the Section 3 is the methods section, describing the details of the model and the theoretical analysis. The Section 4 is the results, divided into basic experiments and comparative experiments, which entailed comparing our model with reinforcement learning models and brain-inspired models to verify the advantages of ours. Section 5 is the discussion, and Section 6 is the conclusion.

## 2. Related Works

Place cells [20] and grid cells [21] have been successively discovered in the mammalian brain and constitute the key factors in spatial cognition and the encoding of instantaneous position in animals [22]. However, these alone are not enough for goal-directed navigation tasks. In order to enable agents to have the ability of path planning like natural creatures, the future state of their space must be predicted and estimated before their movement, which requires a model to establish a connection between the next action and the future position. Phase precession was proposed by O’Keefe and Recce et al. [23], under which before an animal reaches a certain position, the place cells corresponding to the position will be triggered according to the distance to the center of its field, which means that the position cells can encode the expected future position of the animal.

Neurophysiological studies have shown that the hippocampus encodes a short sequence of spatial trajectories from the current location to the target location in rats performing fixed-target location navigation experiments [18]. When the animal is outside the corresponding place field, the place cell can also be activated, which is called “forward scanning” [24]. Erdem et al. proposed a forward-linear advanced-probe navigation model based on trajectory that can predict future paths based on forward-linear advanced trajectory scanning of the agent’s current position [25]. Stachenfeld et al. proposed a hippocampus model of the successor representation (SR) to encode the predicted expressions of the future state of the environment [26]. Cazin et al. proposed a bionic reserve pool computational model that learned to predict the activation of cells at the next location according to delta learning rules [27]. Experiments in rats have found that when subjects are faced with a high-cost choice, they usually pause, and local spikes of neural activity reliably occur in their place cells [28,29]. Ambrose et al. [30] show that the priority remapping of hippocampus place cell sequences is associated with reward. Our model shows similar properties in complex mazes.

Regarding the problem of reward signal attenuation, Mao et al. [31] proposed a path planning algorithm for hierarchical reward diffusion to reduce information loss through the segmentation of environmental states. Jordan et al. [32] proposed a hierarchical path representation that allows agent to perform planning of partial environmental states at a more abstract level. These models can solve the problem of signal attenuation to some extent, but they need to layer the environmental state; the operation is complex, and there is no unified standard; and there is no commonality. In contrast, Khajeh-A et al. [33] proposed a phase-encoding scheme based on a wave propagation algorithm that allows an agent to perform path planning within a single network across multiple spatial scales without the need for hierarchical coding. Huang et al. [34] proposed a brain-inspired model that combines endogenous and exogenous information and ensures the stable propagation of reward signals by adding an olfactory system to the model and using odor as a stable potential energy field. For resources that do not smell, however, such as water, this approach loses its advantages.

Wood et al. found that the modulation of place cell activity depends on the animal’s trajectories through a maze [16]. Other studies have shown that the transient spike sequence generated in animal place cell population is related to its future trajectory, and experiments have shown that the recurrence of place cell sequences is not random and usually reflects the behavioral requirements related to trajectory [18,35]. This transient spike sequence produced by hippocampus place cells is commonly referred to as a replay event [8,14]. Replay events are considered to be the underlying neural mechanism by which the brain converts short-term memory into long-term memory [15]. Shantanu et al. tried to experimentally demonstrate the importance of replay events by electrically stimulating the presence of sharp striations in place cells to disrupt their attendant activity, which resulted in animals taking the wrong route in a spatial memory task [36]. Gupta et al. observed the construction of new pathway trajectories in the rat hippocampus that had never been actually experienced [28]. This is consistent with the virtual path proposed in our model on a biological basis.

From a computational perspective, reinforcement learning frameworks may be suitable for explaining this physiological phenomenon of reactivation [37,38,39,40].

Learning in limited experience [41] is a research hotspot in the field of reinforcement learning, as exploring the environment typically consumes much time and effort [42,43]; therefore, it is important to collect as much knowledge as possible from explored areas for agent learning.

Since the Memory-Replay Mechanism we propose has similarities with experience replay in reinforcement learning, it is introduced and distinguished here.

In 1992, Lin et al. [44] proposed experience-replay, a technique for reusing information gathered from past experiences to improve learning efficiency. A prerequisite for the use of experience-replay is non-policy settings [45]. In non-strategic learning, agents use non-strategic algorithms to interact with the environment and at the same time use objective strategy algorithms to update the value function related to the goal, thus using real experience to collect as much knowledge as possible [46]. However, when the non-strategy algorithm contradicts the operation recommended by the target strategy algorithm, it may lead to poor estimation of the corresponding value function.

Experience-replay in traditional reinforcement learning stores and reuses individual sample points, and after a particular sample replay, the value function is updated, resulting in a change in the corresponding state–action pairs; ideally, the propagation of this change will lead to a large change in the guiding goal of the state–action pairs. The experience-replay algorithm of a single sample point has a relatively small influence on these pairs. However, if it is not a playback of a single sample point, but a replay of a sequence of sample points, the propagation of this signal can be achieved directly. Our algorithm uses this replay mechanism to improve learning efficiency. More replays of this sequence make it possible to more effectively transfer the above effects to other state spaces.

The experience-replay framework developed by Adam et al. [7] involves varied replay experience sequences that are randomly drawn from replay memories. Andrychowicz et al. [47] proposed a hindsight experience-replay algorithm that enables efficient sample learning in reward-sparsity environments. The abovementioned algorithms allow the agent to learn the Q value under the influence of any target and change the agent’s Q table significantly (otherwise, most of the values will remain unchanged in the reward-sparsity environment). Our algorithm also involves the modification of Q values in reward-sparsity environments, and it is well known that animals tend to remember high-reward experiences [48]; therefore, our model is based not on arbitrary target modifications but on TD errors based on sequences of high-reward trajectories.

The experience-replay framework described above is limited to past experience, and all decisions are made on the basis of past experience learning, thereby lacking prediction and judgments of future states.

Inspired by neurophysiological studies such as on the remapping function of hippocampus place cells and the formation of cell assemblies by place cells with frequent coactivity [49], we propose a Memory-Replay Mechanism based on the hippocampus module and the striatum module according to the sequence-replay idea mentioned above. This mechanism not only includes sequence replay but also enables the agent to generate virtual trajectory sequences, and replaying these virtual trajectory sequences can further enhance the agent’s learning efficiency.

As early as 1990, the dyna architecture proposed by Sutton et al. [50] used simulation experience to improve value function estimation, but the simulation experience was generated by reward function and transfer probability models. In contrast, our model collects data directly through the agent interacting with the environment, so that the resulting virtual trajectory sequence is more bio-rational. Fonteneau et al. [51] also proposed an algorithm for generating artificial trajectories, but this algorithm was designed for batch reinforcement learning. In the conversion process from short-term memory to long-term memory, low-reward actions are gradually forgotten. Therefore, our Memory-Replay Mechanism stores the filtered trajectory sequences in internal storage as a memory vault that is constantly updated to ensure that the agent updates the trajectory sequences most relevant to the current task.

## 3. Materials and Methods

**Figure 1:**
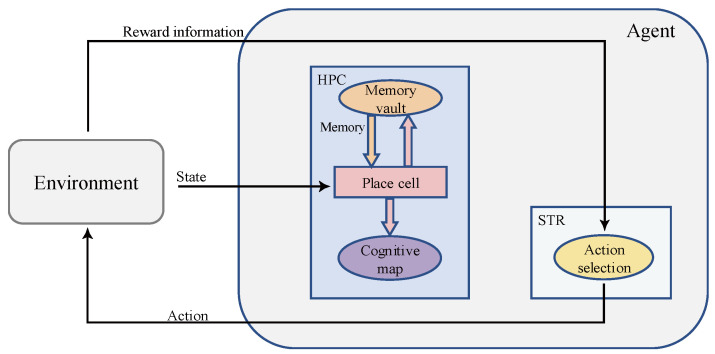
An overall schematic of the model. The square structures represent the anatomical structures that actually exist in the brain, and the oval structures represent the functional structures.

Inspired by pertinent studies on spatial cognition in the brains of animals, we proposed a bionic model to simulate the functions of the rat hippocampus (HPC) and striatum (STR), and the structure of the model is shown in Figure 1. The agent interacts with the environment, continuously obtains the state and reward information of the environment and outputs actions to the environment. The body of the model consists of the HPC and STR. The STR shows a relative preference for action response and reward anticipation [52], and thus, computational models of the STR are often associated with reinforcement learning [18]. In this paper, STR is modeled as an action selection neuron for reward learning. The reward information is transmitted to the HPC module. HPC is the key to spatial cognition, where place cells are the physiological basis for the formation of cognitive maps [20], and the HPC has context-specific memory-encoding patterns that encode the discharge of place cells into sequences [29]. The HPC receives rewards for memory replay and forms a cognitive map. The STR module receives both the cognitive map from the HPC and the reward information from the environment for action selection.

### 3.1. The Hippocampus Module and the Establishment of Single Place Cells

The hippocampus module receives information about the state of the environment and builds an internal cognitive map through the specific discharge of place cells.

**Figure 2:**
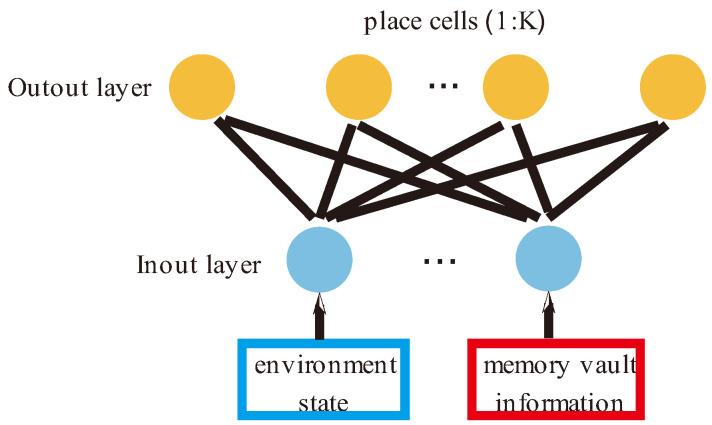
A schematic diagram of a place cell network. The input layer of place cells has two signal sources: One is state information from the environment, and the other is memory information from the hippocampus itself.

In this study, a two-layer feedforward neural network was used to simulate the hippocampus, as shown in Figure 2. The first layer is the input layer, which is responsible for receiving information on the state of the external environment and the internal memory replay signal. Here, we introduce two variables, environment state S and memory vault M, to describe the two kinds of information respectively. The second layer consists of K place cells that are responsible for forming the agent’s cognitive map of the environments. Each place cell receives the input information from the previous layer and is activated, and the activation rate of each neuron is calculated according to Formula (1).

In this paper, place cells are defined as $N\left\{S,M\right\}$, where $S\left(x:y\right)$ represents environmental state information and $M\left(x:y\right)$ represents memory information.

The membrane potential of the place cell $N\left\{S,M\right\}$ is simulated by a Gaussian function, and its specific discharge obeys Equation (1):

(1) $$v_{t}^{i}=\exp(-{\left(\frac{1}{a}\times s_{t}\times \left({\tilde{S}}_{t}\left(x,y\right)-W_{i,S}\left(t\right)\right)+\frac{1}{b}\times m_{t}\times \left({\tilde{M}}_{t}\left(x,y\right)-W_{i,M}\left(t\right)\right)\right)}^{2}/2\sigma_{pc}^{2})$$

where $s_{t}$ indicates the status of the agent’s environment at time t; $m_{t}$ indicates the virtual state of memory vault M at time t; $W_{i,S}\left(t\right)$ represents the connection weight between the ith neuron and the input environment state at time t; $W_{i,M}\left(t\right)$ represents the connection weight between the ith neuron and the input memory vault information at time t; a,b are the dimensions of the vector ${\tilde{S}}_{t}\left(x,y\right),\;{\tilde{M}}_{t}\left(x,y\right)$, a=5,b=1; $\sigma_{pc}\;\mathrm{determines}\;\mathrm{the}\;\mathrm{size}\;\mathrm{of}\;\mathrm{the}\;\mathrm{place}\;\mathrm{field};$ and $0<\sigma_{pc}<1$.

The connection weights between layers are modified with a winner-takes-all strategy. The place cells with the highest firing rate may elect to update the relevant weights. The Equation (2) is as follows:

(2) $$win\left(t\right)=\underset{i}{\mathrm{argmax}\;}v_{t}^{i}$$

The weight of the winning neuron is updated according to Equation (3) and (4):

(3) $$W_{win\left(t\right),S}\left(t+1\right)=W_{win\left(t\right),S}\left(t\right)+\delta \times \left({\tilde{S}}_{t}\left(x,y\right)-W_{win\left(t\right),S}\left(t\right)\right)$$

(4) $$W_{win\left(t\right),M}\left(t+1\right)=W_{win\left(t\right),M}\left(t\right)+\delta \times \left({\tilde{M}}_{t}\left(x,y\right)-W_{win\left(t\right),M}\left(t\right)\right)$$

where $win\left(t\right)$ is the place cell selected at time t and δ is the learning rate (0<δ<1).

The hippocampus module works in a pattern similar to a competitive neural network, matching place fields with place cells through firing information. Cognitive maps are formed by the firing activity of place cells, and the spatial cognition of the environment is formed.

### 3.2. Memory-Replay Mechanisms in the Hippocampus

Our method recognizes the realistic limitations of replay memory [53]. Therefore, we only store a certain amount of information at one time as specified by the internal storage parameter. The generated and selected sequences are stored in replay internal storage as a constantly updated memory vault so that the agent is equipped with the conversion sequences that are most relevant to the task at that time.

It is possible to redefine the memory vault such that high-reward memory and virtual memory correspond to the replay internal storage, and low-reward memory is equivalent to the subconscious, which is only stored and does not participate in memory replay. In this way, the efficiency of replay is improved, and the internal storage consumption of invalid sample sequences in the replay memory vault is reduced.

Let us start by illustrating the storage of trajectory sequences.

The place cell $N\left\{S,M\right\}$ receives the state information $s_{0}\ldots s_{i}$, the reward information $r_{0}\ldots r_{i}$ from the environment, and the internal memory replay information $m_{0}\ldots m_{i}$ from the memory vault. The agent’s action $a_{0}\ldots a_{i}$ will react to the environment, thereby affecting the state of the environment.

A statistical analysis of the peak time of place cells has found that groups of functionally interrelated neurons can form and dissolve on a timescale of tens of milliseconds, and such groups of neurons may also last for a few minutes or more, and the results show that place cells with frequent coactivity tend to form short “cell assemblies” [49].

Therefore, we take the agent from the starting point to the end of this navigation task as a path planning cycle, and the activated place cells are successively recorded in sequence according to the time code of the agent’s own spatial position during the movement process. These elements are integrated into a sequence to simulate the animal’s memory of the path trajectories that have been traveled. We call this set of elements a trajectory sequence Φ as Equation (5).

(5) $$\Phi =\left[N\left\{S\left(x:y\right),M\left(x:y\right)\right\}\;\;\;\;A\left(x:y\right)\;\;\;\;\;R\left(x:y\right)\right]$$

while:

$$S\left(x:y\right)=\left(s_{0}\ldots s_{i}\ldots s_{j}\right)\;M\left(x:y\right)=\left(m_{0}\ldots m_{i}\ldots m_{j}\right)\;A\left(x:y\right)=\left(a_{0}\ldots a_{i}\ldots a_{j}\right)\;R\left(x:y\right)=\left(r_{0}\ldots r_{i}\ldots r_{j}\right)$$

Each time the agent starts from the starting point to the end of the navigation task, a corresponding trajectory sequence will be generated, and the trajectory sequence will be stored in the hippocampus memory vault M as a memory show in Equation (6).

(6) $$M=\left\{\Phi_{1}\ldots \Phi_{i}\ldots \Phi_{j}\right\}$$

The memory vault M will also update continuously as the progress of the agent’s exploration of the environment increases.

The agent interacts with the environment, builds cognitive maps and continuously updates memory vault through state, action and reward signals. The influence of the internal memory replay information from the memory vault on memory vault M itself is not considered here, so we define each state–action–reward $\left\{S\;A\;R\right\}$ as a triple element.

**Figure 3:**
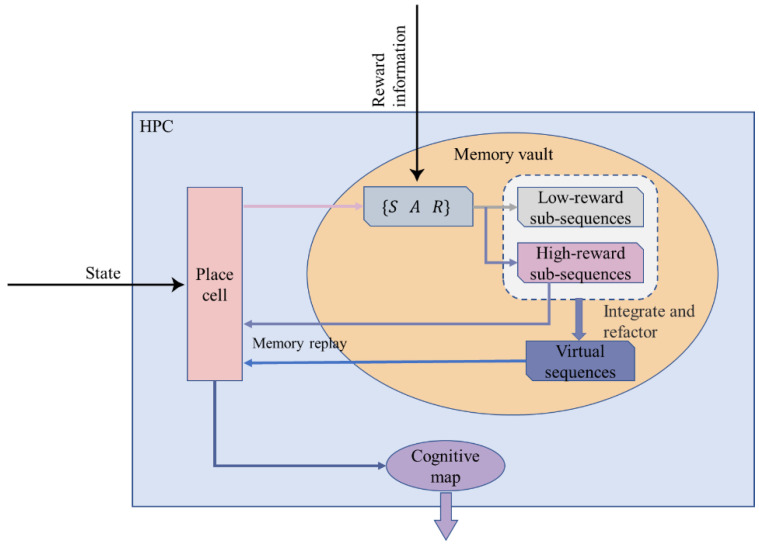
A detailed schematic diagram of the Memory-Replay Mechanism in the hippocampus.

Here we propose a method of updating the memory vault: The agent constructs a virtual path by classifying and integrating the existing paths in the memory vault (that is, the path trajectory that the agent has never actually traveled but only carries out in its brain, which is similar in functional implementation to the forward scan described in Section 1) to improve the learning efficiency of the agent. The schematic diagram of the model is shown in Figure 3.

First, the agent explores an entirely unfamiliar environment and seeks a target reward in the environment. We do not provide the agent with any information about the location of the target reward; rather, it is required to explore the environment and encounter the reward. The agent will be punished if it encounters obstacles during the exploration. If the target reward is not found, the agent also punishes itself because of its own energy consumption.

As the exploration time increases, when the penalty accumulates to a certain threshold, it means that this exploration task has failed, and the current exploration trajectory sequence is defined as a low-reward sequence $\Phi_{L}$, as shown in Equation (7). Since the influence of the memory vault’s internal memory replay information on the memory vault M itself needs to be ignored, we select a subset $\Phi_{Ls}$ of the low-reward sequence $\Phi_{L}$ and store it in the memory vault $M_{L}$ as Equation (9). The mathematical description of low-reward sub-sequences $\Phi_{Ls}$ is shown in Equation (8):

(7) $$\Phi_{L}=\left[N\left\{S\left(x_{l}:y_{l}\right),M\left(x_{l}:y_{l}\right)\right\}\;\;\;\;A\left(x_{l}:y_{l}\right)\;\;\;R\left(x_{l}:y_{l}\right)\right]$$

(8) $$\Phi_{Ls}=\left[S\left(x_{l}:y_{l}\right)\;\;\;A\left(x_{l}:y_{l}\right)\;\;\;R\left(x_{l}:y_{l}\right)\right]$$

(9) $$M_{L}=\left\{\Phi_{L1}\ldots \Phi_{Li}\ldots \Phi_{Lj}\right\}$$

The agent happens to encounter the task target by chance during a certain exploration process, which means the task is successful. The current exploration trajectory sequence is regarded as the high-reward sequence $\Phi_{H}$, as shown in Equation (10). Similarly, we select a subset $\Phi_{Hs}$ of the high-reward sequence $\Phi_{H}$ and store it in the memory vault $M_{H}$ as Equation (12). The mathematical description of high-reward sub-sequences $\Phi_{Hs}$ is shown in Equation (11):

(10) $$\Phi_{H}=\left[N\left\{S\left(x_{h}:y_{h}\right),M\left(x_{h}:y_{h}\right)\right\}\;\;\;A\left(x_{h}:y_{h}\right)\;\;\;R\left(x_{h}:y_{h}\right)\right]$$

(11) $$\Phi_{Hs}=\left[S\left(x_{h}:y_{h}\right)\;\;\;\;A\left(x_{h}:y_{h}\right)\;\;\;R\left(x_{h}:y_{h}\right)\right]$$

(12) $$M_{H}=\left\{\Phi_{H1}\ldots \Phi_{Hi}\ldots \Phi_{Hj}\right\}$$

The agent completes the construction of the cognitive map by exploring the unknown environment many times and then memorizes the paths and trajectories it has experienced in the exploration tasks.

According to neurophysiological studies, the firing activity of place cells in the animal brain mimics path trajectories that they have never actually experienced [29]. Therefore, we propose a method of constructing a virtual path.

In the two path trajectories shown in Figure 6, the blue trajectory begins at the starting point, explores for a period because of the long exploration distance and finally hits the obstacle, resulting in a penalty to the agent and thereby terminating this navigation. The black trajectory, however, also happens to pass through the spatial position mapped by place cell I, and the agent happens to encounter the target reward and obtain a high reward in this trajectory. This triggers the sequence storage and update functionality in the Memory-Replay Mechanism we described earlier. We assume that the black trajectory sequence in Figure 6a is already partially stored in memory vault M. When the agent moves in the blue trajectory from the starting position and reaches the intersection I, the place cell corresponding to the spatial position is activated, and this place cell is the same as the cell contained in the corresponding trajectory sequence stored in the memory vault, which will prompt that trajectory sequence to be reactivated instantaneously, thus constructing an additional trajectory (the generation of the “virtual trajectory sequence”). This virtual trajectory sequence is in turn able to direct the agent towards the target location. As shown in Figure 6b, this green trajectory is a path that the agent has not traveled, and it is constructed using the relevant information about the intersection of parts of the two previously really experienced trajectories, which we call the virtual path.

The virtual path is constructed based on a low-reward sub-sequence $\Phi_{Ls}$ and a high-reward sub-sequence $\Phi_{Hs}$.

In simple terms, first, we assume that there are two trajectories intersecting the same place cell, that is, the place cell is contained by two trajectory sequences, then the two trajectories can be truncated from the place cell and divided into four trajectories. The four trajectories are exchanged and combined to form two virtual path trajectories that the agents have never actually experienced.

Extend the above assumption to the entire memory vault: That is, if øxy and $øx^{\prime }y^{\prime }$ are sets containing all the elements of sequences $N\left\{S\left(x:y\right),M\left(x:y\right)\right\}$ and $N\left\{S\left(x^{\prime }:y^{\prime }\right),M\left(x^{\prime }:y^{\prime }\right)\right\},\;$respectively, and there is any$\;I\in \left\{øxy⋂øx^{\prime }y^{\prime }\right\}$, then I is the set of intersection points of the trajectory sequence Φ and $\Phi^{\prime }$.

In order to enable the agent to find the target reward more quickly, the constructed virtual path has to be strongly correlated with the reward information, so we need the end point of the virtual path to point to the location of the target reward. The low-reward sub-sequence $\Phi_{Ls}$ and the high-reward sub-sequence $\Phi_{Hs}$ are decomposed at each intersection to obtain Equations (13) and (14):

(13) $$\Phi_{Ls}=\left[\begin{array}{c} \Phi_{Ls}^{1}\\ \Phi_{Ls}^{2} \end{array}\right]$$

(14) $$\Phi_{Hs}=\left[\begin{array}{c} \Phi_{Hs}^{1}\\ \Phi_{Hs}^{2} \end{array}\right]$$

The combinations can be rearranged to form two “new paths”:

(15) $$\Phi^{\prime }=\left[\begin{array}{c} \Phi_{Ls}^{1}\\ \Phi_{Hs}^{2} \end{array}\right]$$

(16) $$\Phi^{\prime \prime }=\left[\begin{array}{c} \Phi_{Hs}^{1}\\ \Phi_{Ls}^{2} \end{array}\right]$$

From Equation (16), it can be seen that the second half of this new path $\Phi^{\prime \prime }$ coincides with the second half of the low-reward sub-sequence $\Phi_{Ls}$, that is, the location of the reward cannot be reached in the end, so we will only select the new path $\Phi^{\prime }$ shown in Equation (15) for incorporation into the memory vault as an effective memory.

(17) $$\Phi^{*}=\left[\begin{array}{c} \Phi_{Ls}^{1}\\ \Phi_{Hs}^{2} \end{array}\right]$$

And the virtual trajectory sequences are stored in the hippocampus memory vault $M^{*}$ as Equation (18).

(18) $$M^{*}=\left\{\Phi_{1}^{*}\ldots \Phi_{i}^{*}\ldots \Phi_{n_{v}}^{*}\right\}$$

The trajectory sequences $\Phi_{Hs}$ and $\Phi^{*}$ described by Equations (11) and (17) are both part of the memory vault M, and are associated with high rewards, and both will be preferentially selected during memory replay where $\Phi^{*}$ is the integrated virtual path and includes $\Phi_{Ls}^{1}$ and $\Phi_{Hs}^{2}$, which are subsets of $\Phi_{Ls}$ and $\Phi_{Hs}$, respectively. 

**Figure 4:**
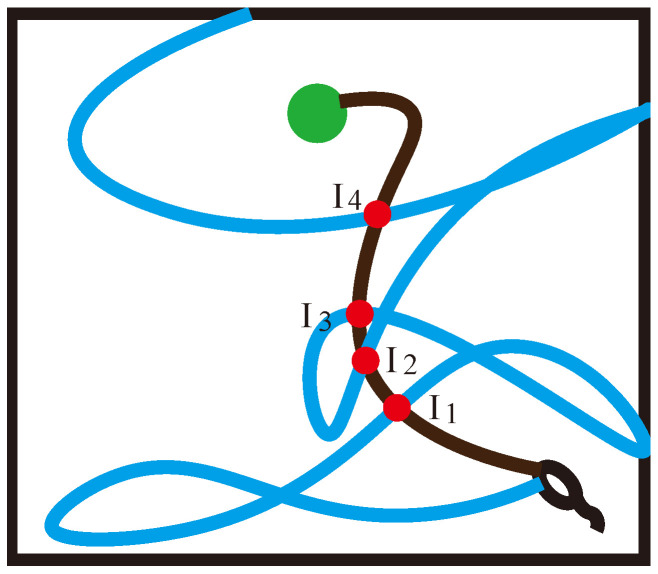
Schematic diagram of multiple intersections. $I_{1},I_{2},I_{3},I_{4}$ are the intersection points of the low-reward sequence $\Phi_{L}$ and the high-reward sequence $\Phi_{H}$ in different spatial locations respectively.

When $\Phi^{*}$ is replayed, high-reward-related information is propagated from $\Phi_{Hs}^{2}$ to $\Phi_{Ls}^{1}$. Therefore, if there are multiple intersections $\left\{I_{1},I_{2}\ldots I_{j}\right\}$ in the path trajectory, as shown in Figure 4, we prefer $I_{4}$. Since we will not give the agent any hint about the reward position during the experiment, the number of high-reward sub-sequences $\Phi_{Hs}$ will be much lower than the number of low-reward sub-sequences $\Phi_{Ls}$. To increase the level of agent exploration and the effectiveness of environmental exploration, if there are multiple intersections, we choose to focus on the low-reward sub-sequence $\Phi_{Ls}$ and choose the point with the longest $\Phi_{Ls}^{1}$. That is, if there are multiple overlapping place cells in the low-reward sequence $\Phi_{L}$ and the high-reward sequence $\Phi_{H}$, when the path decomposition is performed, the intersection points $I_{j}$ at the end of the low-reward sub-sequence $\Phi_{Ls}$ are selected as the sequence decomposition points, so that Equation (19) can be obtained:

(19) $$\Phi_{Ls}=\left[\begin{array}{c} \Phi_{Lsmax}^{1}\\ \Phi_{Lsmin}^{2} \end{array}\right]$$

This Memory-Replay Mechanism will enrich the memory vault $M^{*}$ of the virtual trajectory sequence so that the state–action value undergoes a large number of improvements, thereby reducing the probability of local optima.

Each state–action–reward triple $\left\{S\;A\;R\right\}$ in the high-reward sub-sequence $\Phi_{Hs}$ and the virtual trajectory sequence $\Phi^{*}$ described above is replayed in the place cells, as if the agent were actually experiencing them again. Replay in accordance with the standard Q-learning update as Equation (20):

(20) $$Q\left(s_{j},a_{j}\right)\leftarrow Q\left(s_{j},a_{j}\right)+\alpha \left[R\left(s_{j},a_{j}\right)+\gamma \underset{a^{\prime }}{\underset{︸}{max}}Q\left(s_{j+1},a^{\prime }\right)-Q\left(s_{j},a_{j}\right)\right]$$

where Q and R represent the action value function and reward function, respectively; $a^{\prime }$ represents any action in the action set.

The hippocampus continuously replays the trajectory sequences in memory vaults $M_{H}$ and $M^{*}$, and the cognitive map continuously corrects spatial cognition under the stimulation of memory replay.

This artificially constructed sequence of virtual trajectories offers the agent considerable possibilities for learning progress because when reactivation occurs, this mechanism helps the agent to fully propagate the correlation of the target (characterized by a large number of levels of TD error) to other regions of the state-action spaces. These reactivations and memory updates complement and perform updates to the value functions, thus accelerating the learning of related objectives.

### 3.3. The Benefits of Incorporating a Memory-Replay Mechanism

First, the Memory-Replay Mechanism proposed in this paper is a brain-inspired algorithm. Before an animal faces a major decision in a spatial navigation task or during a rest period, its place cells will exhibit a phenomenon of regular reactivation. Studies have shown that this reactivation of the hippocampus is positively correlated with the progress of the learning rate in animals, and hippocampus reactivation was also associated with future expectation encoding and reward location preference. Our algorithm has a sound neurophysiological basis.

**Figure 5:**
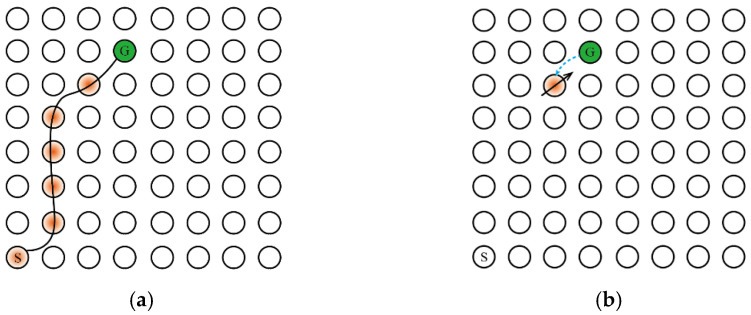

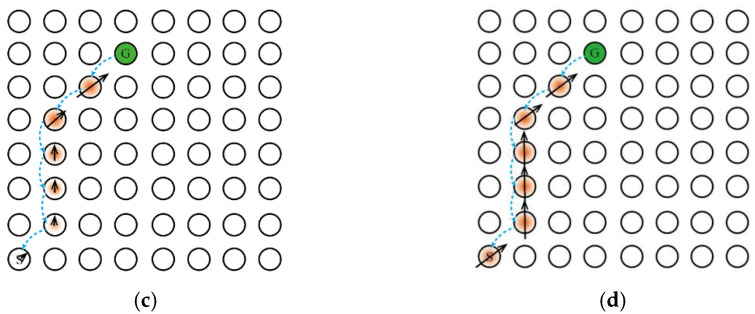
The Memory-Replay Mechanism can replace qualification tracking without parameter tuning. The direction of the black solid arrow represents the action selection made by the agent to obtain the corresponding reward information, and the length represents the information amount of the reward signal returned from the previous state; the blue dashed arrow represents the transmission direction of the reward information between the place cells. (**a**) The optimal path trajectory found by the agent. (**b**) Reward signaling in the absence of qualification tracking. (**c**) Reward signaling with qualification tracking, parameter λ < 1, the amount of information transmitted back to the reward signal from the previous state decays exponentially. (**d**) Reward signaling by Memory-Replay Mechanism, that is, with qualification tracking, parameter λ = 1. The return of the reward signal from the previous state has no loss throughout the trajectory (under ideal conditions).

Second, the addition of the Memory-Replay Mechanism has other benefits, For the sake of illustration, we will use the simple maze problem shown in Figure 5a. The agent needs to take the shortest path to reach the goal position from the starting point and only receives a positive reward when it reaches the goal position. The circle in Figure 5a represents the place field corresponding to the place cell in the environment and the hippocampus cognitive map, the orange circle represents the activation of the place cell corresponding to the place field, S represents the starting position, G represents the goal, and the black curve represents the agent’s movement track in the environment.

One of the benefits of memory replay is that it can achieve a similar effect with qualification tracking. Assuming that the agent has traveled the trajectory shown in Figure 5a and uses a temporal-difference incremental learning method, such as Q-learning, we set the learning rate α to 1 to maximize the dissemination of information. If qualification tracking is not added in the process of information transmission, when the agent reaches G, the received reward signal can only transmit to the previous state of G, as shown in Figure 5b. When joining qualification tracking and parameter λ < 1, the reward signal propagates along the trajectory from the target position to all states of the starting position, as shown in Figure 5c. When the parameter λ = 1 (ideally), the reward signal is appropriately discounted to propagate to all states on the trajectory, as shown in Figure 5d. Returning to the Memory-Replay Mechanism in this paper, it can reactivate the place cells on the path trajectory multiple times in accordance with the place cell activation sequence, which will make the reward signal propagate along the trajectory from the goal location to all states of the starting location. As the number of memory replays increases, the result of reward signal transmission can be similar to the result obtained when the qualification tracking parameter is set to 1 in Figure 5d. Moreover, the Memory-Replay Mechanism can achieve the optimal reward signal transmission effect without adjusting parameters.

**Figure 6:**
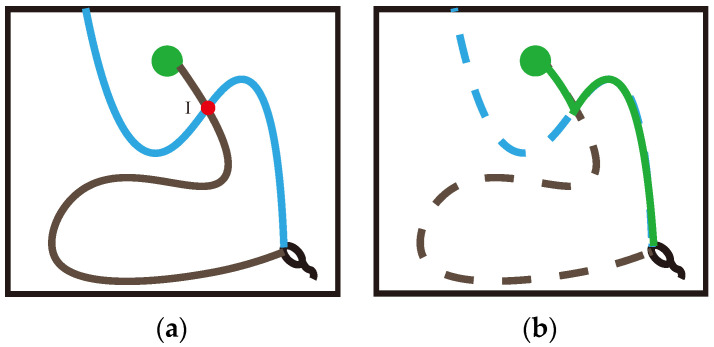
A schematic diagram of one intersection: (**a**) the two trajectories recorded in memory vault M during the agent’s exploration of the environment; the blue trajectory is the low-reward trajectory, the black trajectory is the high-reward trajectory, and the two trajectories intersect at point I. (**b**) According to the Memory-Replay Mechanism we described, using the intersection as the dividing point and two real experienced trajectory sequences as the basis, construct a virtual path that the agent has never navigated.

Another advantage of the Memory-Replay Mechanism is that it can split and integrate multiple track sequences to build virtual paths. Again, we use a simple maze experiment as an example to illustrate: Assuming that the agent has walked through the two paths trajectories in Figure 6a, if we use the Q-learning algorithm with qualified tracking, only when the agent moves in accordance with the black path trajectory in Figure 6a can it receive the reward signal returned from the goal position. In contrast, the virtual path constructed by the Memory-Replay Mechanism, such as the solid line shown in Figure 6b, has not been actually traversed by the agent, but the reward signal of the goal position can still be received from the path.

Algorithm 1. The Memory-Replay Mechanism.
**Algorithm 1. Memory-Replay Mechanism****1:****inputs:** Trajectory sequence of high-reward $\Phi_{H}$
   Trajectory sequence of low-reward $\Phi_{L}$

   Memory vault of hippocampal $M^{*}$ for storing the virtual trajectory sequence**2:****Initialize:** parameters: $N_{iter}$, learning rate, discount factor**3:****for** h = $p\cdot N_{iter}$
**do****4:**  find the intersection of the trajectory sequence of high-reward $\Phi_{Hs}$ and the trajectory sequence of low-reward $\Phi_{Ls}$**5:**  use intersection I to store the constructed element $I\in \left\{ø\mathrm{xy}⋂øx^{\prime }y^{\prime }\right\}$**6:**  **if**
I is not ∅,**7:**   **for**
i⊂I
**do****8:**regarded i as the intersection point**9:**decompose $\Phi_{Ls}$ and $\Phi_{Hs}$ as the Equations (8) and (11)**10:****end****11:****end****12:**construct the virtual trajectory sequence $\Phi^{*}$ as the Equation (15)**13:**store them in the memory vault of hippocampal $L^{*}$**14:****end****15:****for** k = 1 : $n_{v}$
**do****16:**  as Equation (16), $n_{s}=$ the number of $\left\{s\;\;a\;\;r\right\}$ triads in$\;\Phi^{*}$**17:**j=1**18:****While**$j\leq n_{s}$**do**
$Q\left(s_{j},a_{j}\right)\leftarrow Q\left(s_{j},a_{j}\right)+\alpha [R\left(s_{j},a_{j}\right)+\gamma \underset{a^{\prime }}{\underset{︸}{max}}Q\left(s_{j+1},a^{\prime }\right)-Q\left(s_{j},a_{j}\right)]$**19:**   j=j+1
**20:** **21:**   **end** **end**

### 3.4. Striatum Model

In neurophysiological studies, it has been shown that the striatum is involved in reward learning and function in selecting action [52]. Therefore, this study uses action neurons as the basis of the striatum model. Computational models of the striatum are often associated with reinforcement learning and TD learning [18], that is, reward information from the environment, which is quantified with Equation (21):

(21) $$R_{t}=\left\{\begin{array}{l} -100\\ 100\\ -10 \end{array}\right.\;\begin{array}{c} if\;there\;is\;a\;obstacle\\ if\;there\;is\;the\;goal\\ otherwise \end{array}$$

**Figure 7:**
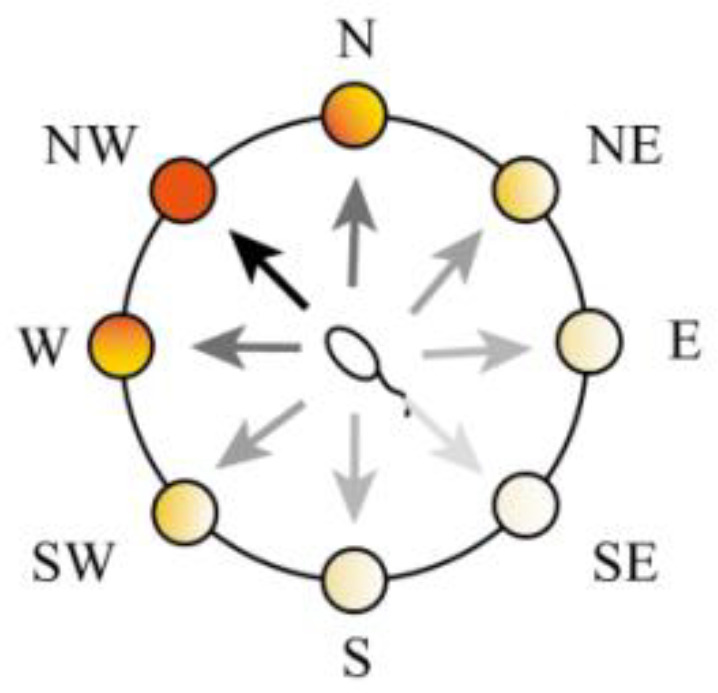
A schematic diagram of the action neurons.

**Figure 8:**
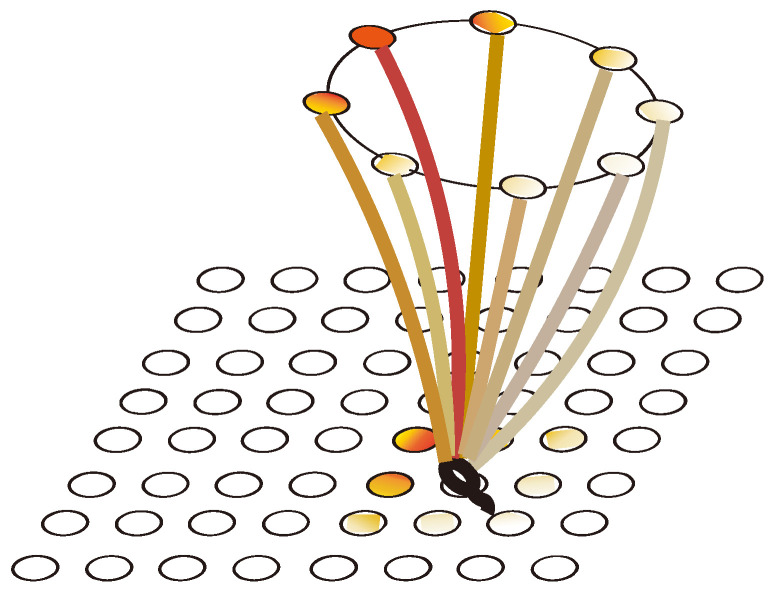
The mapping relationships between cognitive maps and action neurons.

The action selection neuron group consists of eight groups of cells as shown in Figure 7, where each group of cells represents an action: east (E), west (W), south (S), north (N), southeast (SE), northeast (NE), southwest (SW), and northwest (NW). Each group of action-selecting neurons receives place cell weights from the hippocampal cognitive map and reward information as shown in Figure 8.

In this paper, we use temporal-difference learning to calculate the correlation between place cell discharge and reward information. The replay of the memory will reinforce the TD error information contained in the sequence and finally will make the estimate of the value function more accurate; then, the cognitive map construction will be more suitable for the actual spatial environment. Replay is performed according to the Q-like learning update equation shown in Equation (22):

(22) $$Q\left(s_{j},a_{j}\right)\leftarrow Q\left(s_{j},a_{j}\right)+\alpha \left[R\left(s_{j},a_{j}\right)+\gamma \underset{a^{\prime }}{\underset{︸}{max}}Q\left(s_{j+1},a^{\prime }\right)-Q\left(s_{j},a_{j}\right)F_{i}\left(v_{t}\right)\right]$$

Among them, Q and R represent the action value function and reward function, respectively, $a^{\prime }$ represents any action in the action set, and $F_{i}\left(r_{t}\right)$ is the position cell filter, as shown in Equation (23).

Action-selection neurons perform actions under the guidance of cognitive maps. In the initial state, the cognitive map is initialized, $Q\left(s_{j},a_{j}\right)=0$; at this time, the agent randomly selects an action with probability P or keeps the current direction and continues to move forward one unit with probability 1−P. When $Q\left(s_{j},a_{j}\right)\neq 0$, the agent selects an action using an ε− greedy strategy; that is, the agent selects the action that maximizes Q according to the cognitive map with a probability of 1−ε or moves randomly with a probability of ε (0<ε<1).

In order to better represent the mechanism of action selection, we established a filter to select the group of action neurons with the most active firing signals as shown in Equation (23):

(23) $$F_{i}\left(v_{t}\right)=\left\{\begin{array}{cc} 1 & v_{t}^{i}>\theta \\ 0 & otherwise \end{array}\right.$$

where θ is the threshold of the filter 0<θ<1.

After this action selection, the connection weights between the place cells and the action selection neurons are updated according to the value function formula shown in Equation (24):

(24) $$Q\left(s_{j},a_{j}\right)=\frac{\sum_{i}Q\left(s_{j},a_{j}\right)F_{i}\left(v_{t}\right)}{\sum_{i}F_{i}\left(v_{t}\right)}$$

In our model, the value function is scattered in the network of the cognitive map and the connection weights between neurons, and multiple place cells may be involved in one update. At the same time, a filter is introduced to reduce the number of place cells involved in the update, which further improved the efficiency of action selection.

## 4. Results

The Morris water maze (MWM) experiment was invented by the British biologist Morris in 1984. It is a widely recognized classic experiment that has been widely used in learning and memory, hippocampus, neurophysiology and behavioral research, etc. It is mainly used to test the learning and memory ability of subjects for sense of direction (spatial positioning). Therefore, we first simulated the Morris water maze experiment to prove that our model has spatial learning and memory ability. We then changed the location of the survival platform and the starting point to test the suitability of the model for the environment. Next, we added different obstacles to the environment to test the robustness of the model.

In the comparative experiments section, we compared two traditional reinforcement learning navigation models with our model to illustrate the advantages of our model in terms of environmental exploration rate and learning efficiency. In addition, we also conducted comparative experiments with two types of brain-inspired models.

Since Huang et al. [34] and Khajeh-Alijani et al. [33] also focused on solving the problem of the attenuation of neuronal signals, similar to our model, there was no need to perform any hierarchical treatment of environmental states, so we chose to compare experiments with their models. Khajeh-Alijani et al. designed a complex maze to test the signal transmission strength of the model. We reproduced this complex maze experiment and compared the experimental results.

### 4.1. Parameter Settings

**Figure 9:**
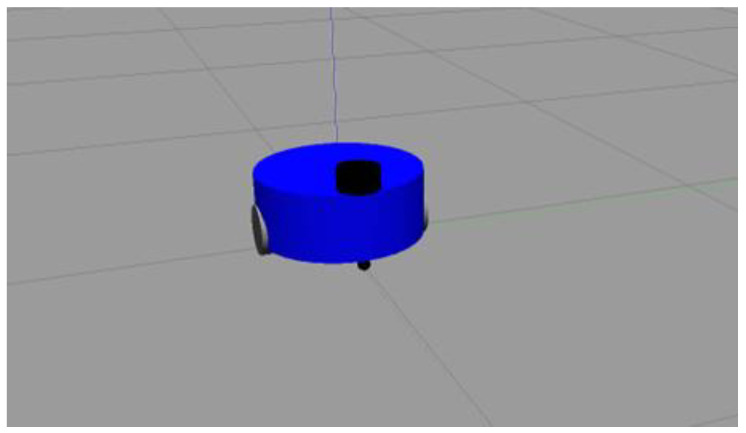
Robot model in 3D environment.

In the simulation experiment, the space is 20 × 20 square, and the agent is placed in a random position in the environment. Its target task is to find the hidden survival platform in the environment. The agent is modeled in a 3D environment as shown in Figure 9. In this study, the agent is equipped with a lidar, which can search for an area with a radius of one unit around it and can detect when it has “hit” an obstacle. It can move through the environment at a maximum speed of 1 unit per time step by performing actions.

The agent can choose between eight directions of action, east, south, west, north, northeast, southeast, northwest, and southwest, and of course, they can choose to remain where it is. The choice of these actions is probabilistic, and in the path-planning process, the probability of the expected action actually being executed is 80%%. When the remaining 20% probability occurs, the actual execution of the action may deviate by 1 unit in the other direction based on the expected action. If the agent chooses an action that causes it to hit an obstacle, then its position will be kept as constant as possible; otherwise, it will receive a high penalty (−100). The agent adopts a relatively greedy strategy, setting the ε = 0.1 in order to maximize the expectation of rewards. If the agent reaches the specified target position, it receives a high reward (100). In addition to this, due to the consumption of its own energy during navigation, each action choice that fails to reach the target position receives a slight penalty (−10). In all experiments, the agent’s learning rate α = 0.3 and the discount factor γ = 0.9. The parameter settings for the model are shown in Table 1.

### 4.2. The Simpel Experiment of Morris Water Maze

**Figure 10:**
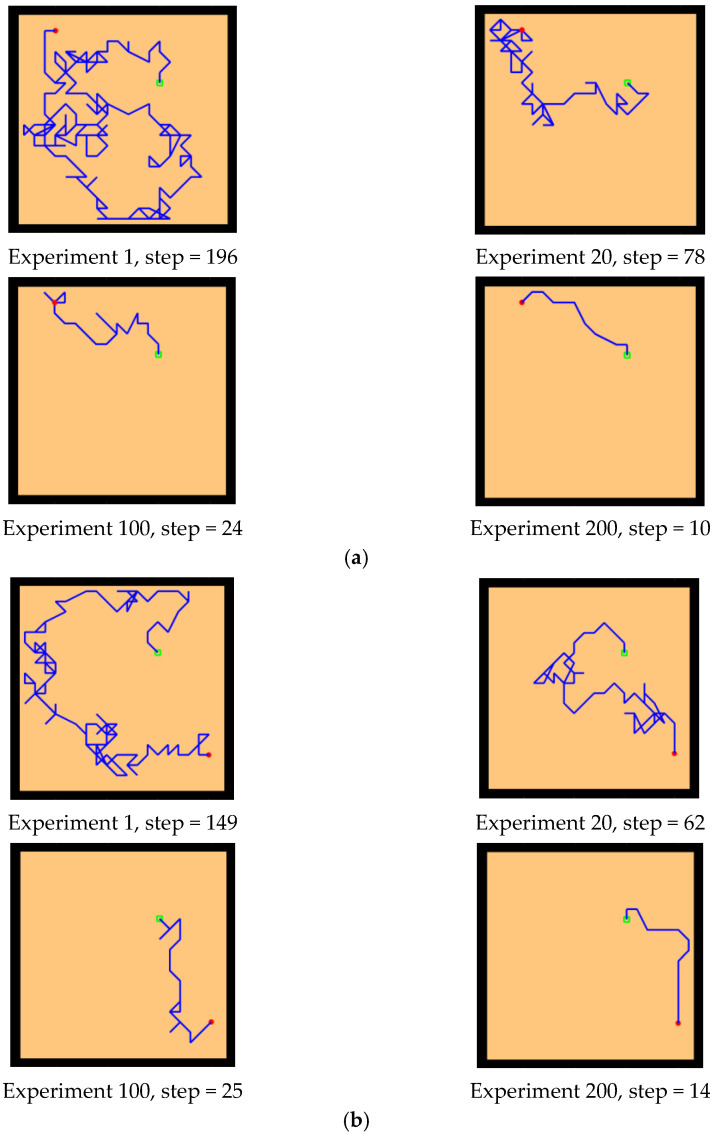
Basic experiment. The red circle is the starting position of the agent, the green square is the position of the hidden survival platform, and the blue line is the moving trajectory of the agent. The position of the starting point is changed, and the position of the survival platform remains unchanged. Two groups of experiments (**a**,**b**): (**a**) starting point a, (**b**) starting point B.

**Figure 11:**
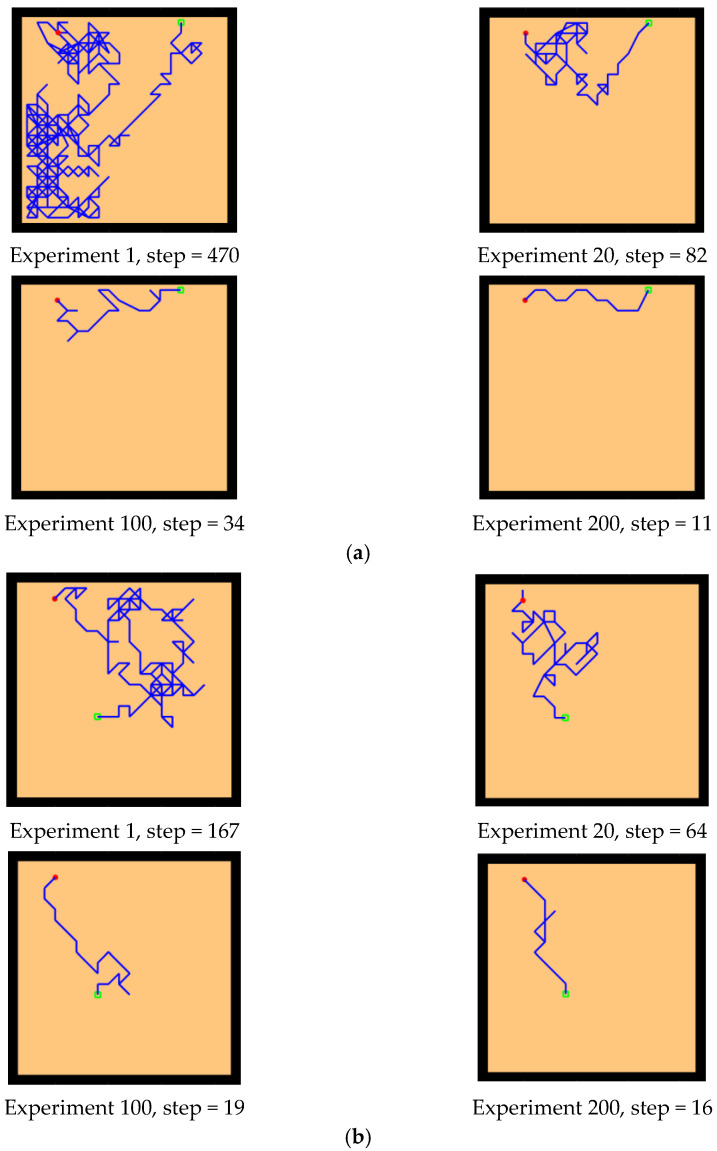
Basic experiment. The position of the starting point remains unchanged, and the position of the survival platform changes. Two groups of experiments: (**a**,**b**). (**a**) survival platform A, (**b**) survival platform B.

Basic experiments to verify the validity of the model:

First, we conducted preliminary experiments to simulate the Morris water maze environment to verify the effectiveness of our model. By changing the position of the starting point and the location of the survival platform, we tested the adaptability and robustness of our model. The Morris water maze experiment consists of a pool and a movable survival platform hidden under the water surface. The experimental environment is a 20 × 20 square space. The experimental results are shown in Figure 10 and Figure 11, which record the paths of the agent from the starting point to the survival platform.

First, the experiments showed that our model can simulate the behavior of rats in the Morris water maze and can successfully find a survival platform after exploring the environment, which proves that the agent has the ability of environmental cognition. Second, our model is an unsupervised learning model, without any supervision or instruction to the agent during the whole exploration process. Finally, our model is adaptive. After changing the starting position and the position of the survival platform, the agent can still successfully reach the survival platform, which indicates that our model is goal-oriented and can better adapt to new environmental conditions compared with the task-oriented model. In addition, our model exhibits the characteristics of progressive learning. At the beginning of the experiment, it usually takes more exploration time and a larger step size to complete the task. With the continuous learning of the environmental state, it can finally reach the survival platform with a shorter path. This is consistent with the movement of animal environmental exploration described by Thorndike et al. [54].

**Figure 12:**
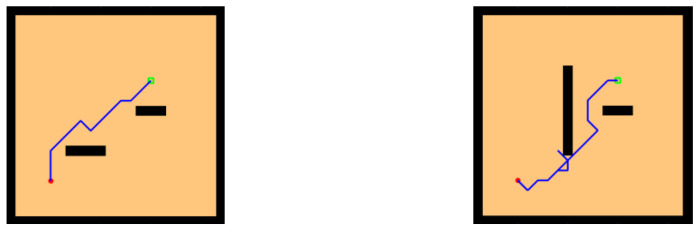
Water maze experiment with the simple obstacles.

To verify the robustness of the model, we added some obstacles to the water maze beginning with adding some simple block-like obstacles between the starting point and the survival platform, as shown in Figure 12. The agent sensed the presence of obstacles through lidar and detours, and the chosen path was relatively short.

**Figure 13:**
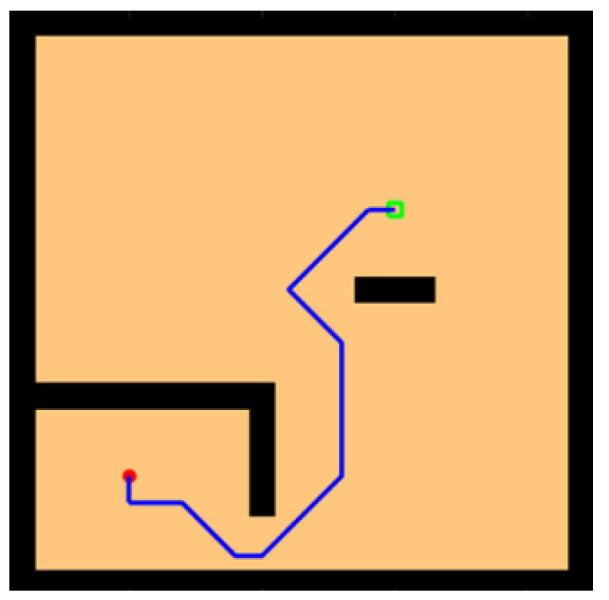
Water maze experiment with the U-shaped obstacles.

In environments with sparse rewards, local optimality is a tricky problem, so we designed a U-shaped obstacle to test whether the model would be trapped inside the U shape. As shown in Figure 13, our model successfully found a path out of the U-shaped obstacle and reached the survival platform in a shorter path.

### 4.3. Comparative Experiment

In this module, we compare our model with reinforcement learning models and other brain-inspired models to test the spatial cognitive efficiency of our model and its solution to the problem of signal attenuation.

#### 4.3.1. Comparison with the Reinforcement Learning Model

The introduction of reinforcement learning in the navigation model can reflect the animal exploratory behavior described by Thorndike et al. from a machine learning perspective [54]. Here, we compare the Q-learning model and the reinforcement learning with uniform random sampling experience-replay model with our model in the water maze setting. Q learning is a model-free reinforcement-learning algorithm proposed by Watkins that is essentially a Markov decision process (MVP) [55]. By continuously interacting with the environment and trying to record a series of previous decision-making processes, the action with a larger score in the record is selected with a higher probability during a decision-making process.

The environmental setting of the comparison experiment is the same as the basic experiment. After several experiments, the parameters of Q learning are set as follows: α=0.3, γ=0.9, λ=1 where α is the learning rate, γ is the discount factor and λ is the attenuation factor of signal tracking. The environmental setup for all comparative experiments is the same as a 20 × 20 state space, the action space has eight action choices, and the reward function is also the same as our model.

**Figure 14:**
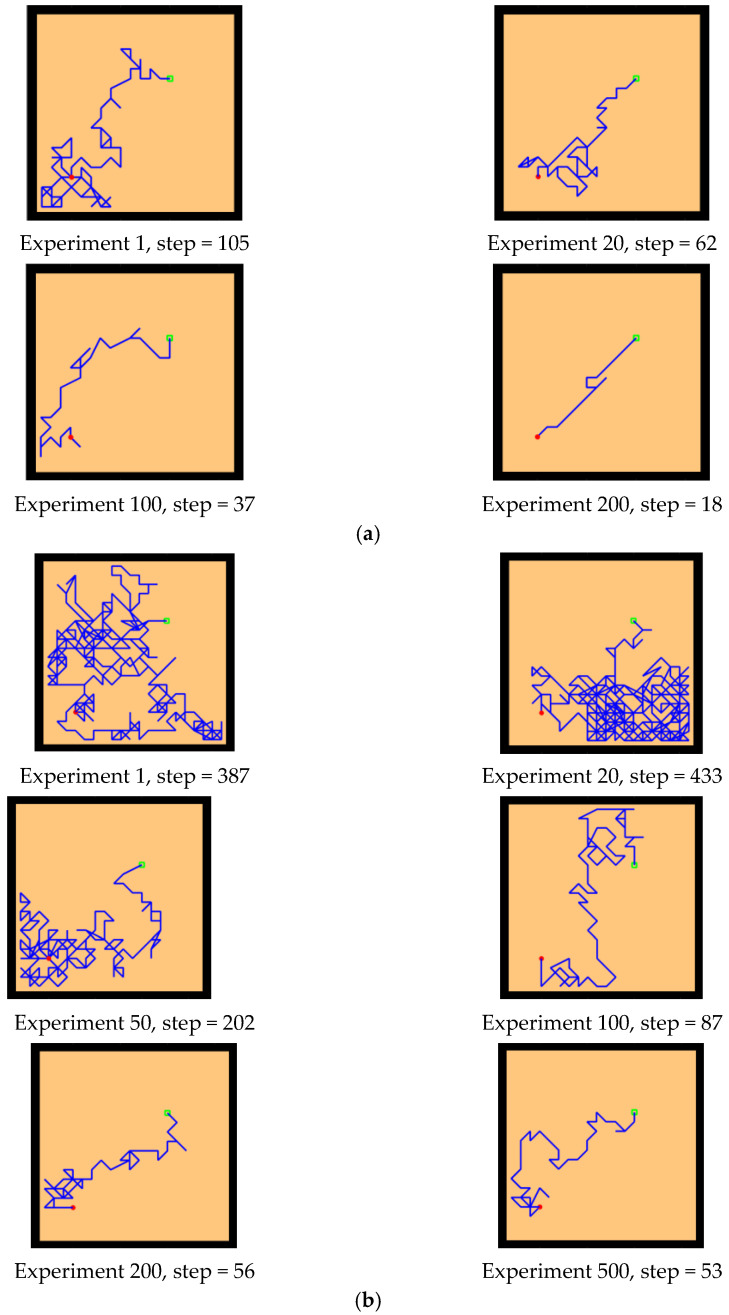
Comparative experiment of the Q-learning model and our model in the water maze. (**a**) Our model. (**b**) Q-learning model.

The experimental setups of the Q-learning algorithm and our model are shown in Figure 14. Both models can enable the agent to successfully find the survival platform. However, it can be seen from the results that the agent with the navigation of the Q-learning algorithm is not sensitive to the position of the survival platform and cannot judge the orientation after quickly approaching the position of the platform. In contrast, our model is faster and takes a shorter path to reach the survival platform.

**Figure 15:**
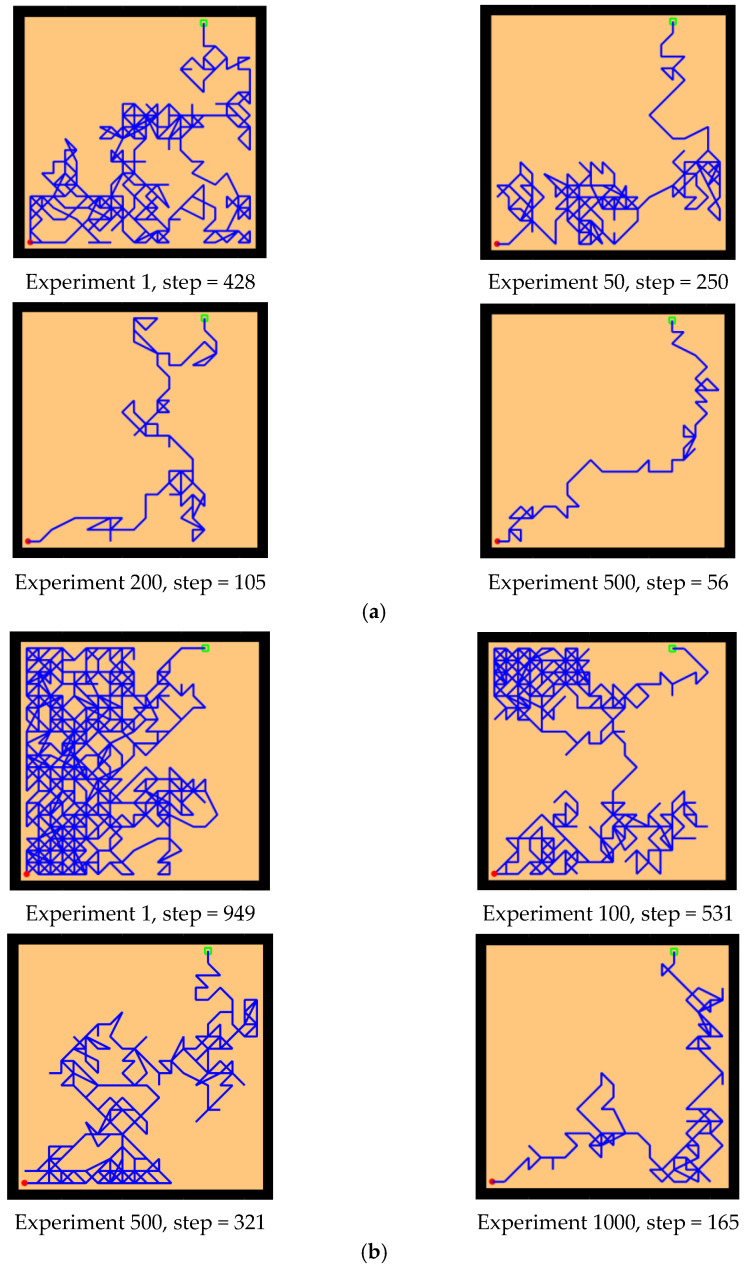
Further comparative experiment with the Q-learning model and our model in the water maze. (**a**) Our model. (**b**) Q-learning model.

Next, we changed the starting position of the agent and the position of the survival platform, and we expanded the distance between them to test the adaptability of the model in the environment. As we can see from the experimental results in Figure 15, after changing the starting position and the hidden platform position, our model can still maintain robustness, while the agent controlled by the Q-learning needs more training time to reach the survival platform smoothly. In Figure 15a, the agent using our model can reach the survival platform smoothly after 200 rounds of experiment, and the efficiency can be improved by increasing the number of experiments. The agent using Q-learning in Figure 15b can reach the survival platform relatively smoothly after 1000 experiments.

The Memory-Replay Mechanism proposed in this model is similar to the experience-replay model in reinforcement learning. Therefore, in addition to the Q-learning model, we also chose a reinforcement learning model with uniform random sampling experience replay for comparative experiments. To make the results easier to observe, we increased the sensitivity of the agent to rewards and penalties, and we modified Equation (18) to Equation (25):

(25) $$Rt=\left\{\begin{array}{cc} -500 & if\;there\;is\;a\;obstacle\\ 1000 & if\;there\;is\;the\;goal\\ -10 & otherwise \end{array}\right.$$

**Figure 16:**
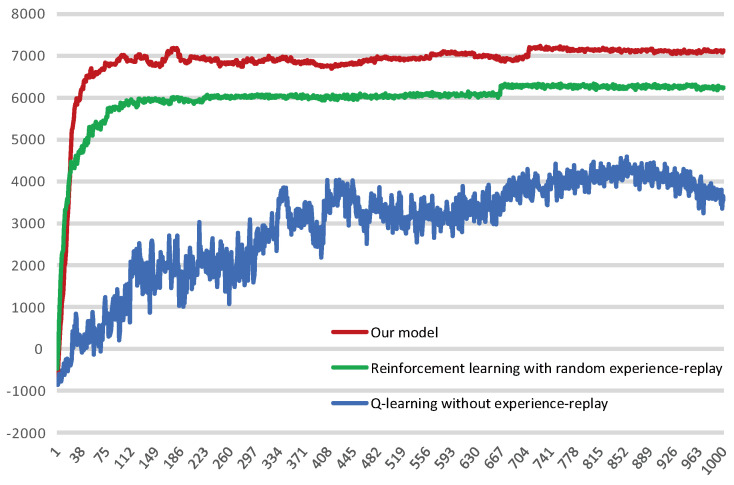
Average reward obtained by the agent per navigation. The red curve represents our model, the green curve represents reinforcement learning with random experience replay and the blue represents Q-learning without experience-replay.

Figure 16 shows the average reward intensity obtained by the agent for each navigation. Although the reinforcement learning model with random experience replay shows faster learning efficiency in the initial learning, our model can maintain this high learning rate for a longer time in the process of exploring, and compared with the random experience-replay reinforcement learning, our model can obtain a higher average reward. This shows that our model can find a shorter navigation path, and the probability of falling into a local optimum is lower.

We intercepted some of the data for the stabilization interval and calculated their averages separately, as shown in Table 2. The results show that the average reward our model received is 14.118% higher than the reinforcement learning with random experience replay. Of course, the magnitude of the increase is related to the fact that we changed the reward value for reaching the survival platform, but our model did receive a higher average reward compared with these two reinforcement learning models.

**Figure 17:**
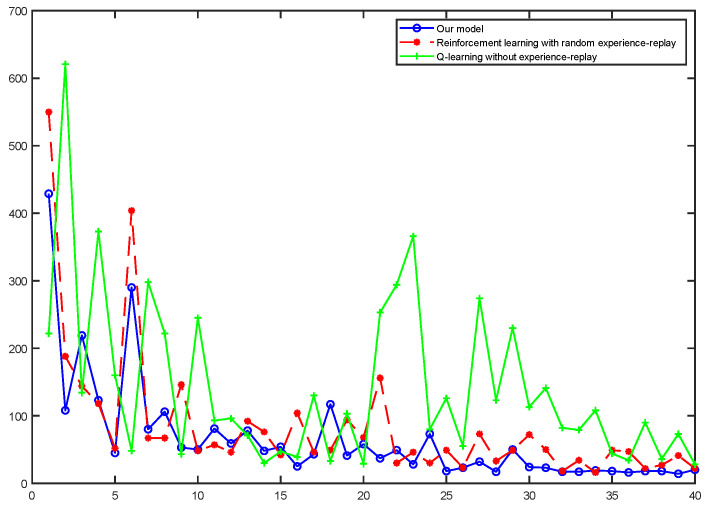
Comparison of steps of our model with two reinforcement learning models for a water maze experiment under the same conditions: The solid blue line represents our model, the red dotted line represents the reinforcement learning with random experience replay and the solid green line represents the Q-learning without experience replay.

At the same time, we counted the numbers of steps to reach the survival platform in the water maze experiment in the three models, as shown in Figure 17. The results show that the step count of the Q-learning without experience replay was extremely unstable, indicating that its learning efficiency was low, and it was unable to learn the state of the environment and choose the appropriate route in a limited number of trainings. Our model explores and learns the environment more thoroughly in the early stages and would quickly able to stabilize the number of steps at a value. Reinforcement learning with random experience replay can also lock in targets relatively quickly in terms of path selection, but its stability is not as good as our algorithm.

The following experiments illustrate the solution of our model to the signal attenuation problem and compare our model with the random experience-replay reinforcement learning algorithm. The experimental environment is still a 20 × 20 water maze, the agent starts from the lower left corner and the target task is to reach the blue survival platform in the upper right corner with a shorter path.

**Figure 18:**
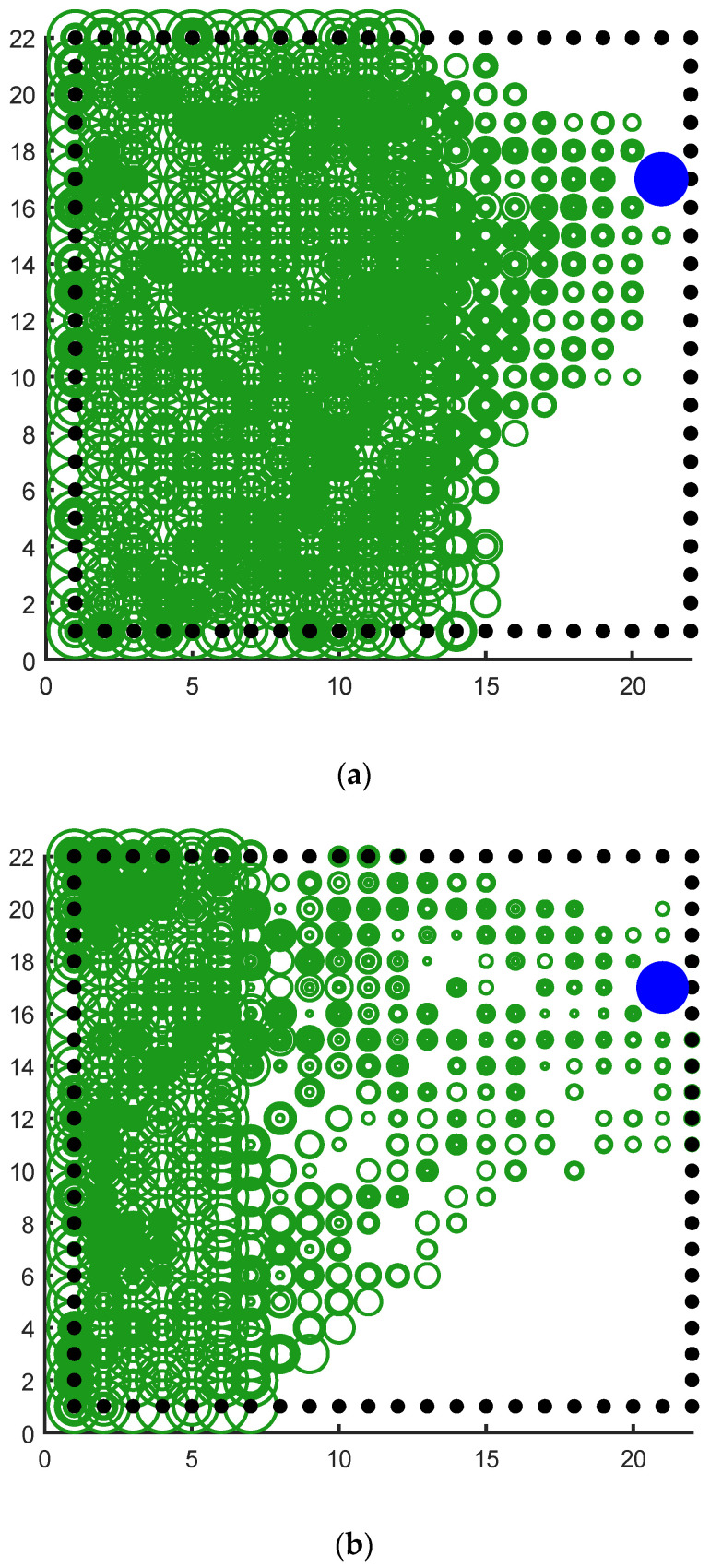
Agent starts from the lower left corner, the blue circle is the survival platform and the black circle is the obstacle. The size of the radius of the green ring indicates the strength of the position information returned by the place cell corresponding to the environmental position of the ring received by the agent at the starting position. The larger the ring radius, the higher the strength of the returned position information. The width of the ring represents the reactivation times of the place cells corresponding to the environmental location, and the larger the ring width, the higher the reactivation times. (**a**) The signal propagation diagram of our model. (**b**) The signal propagation diagram of the reinforcement learning with random experience replay.

It can be seen clearly from Figure 18a that the green rings show a direction trend from the starting position in the lower left corner to the position of the blue survival platform in the upper right corner, and the green rings have roughly completed the coverage of the environment. This indicates that the agent has a high degree of exploration in the environment. This result shows that the agent using our model not only has a high degree of memory for the location of the survival platform but also has a high degree of exploration of the environment. A higher degree of exploration means that the probability of falling into a local optimum is smaller. Figure 18b shows the signal propagation diagram of the reinforcement learning with random experience replay. Compared with Figure 18a, it is obvious that the size of the green ring on the right side of the two figures is different from the starting position of the agent. The radius of the right rings in Figure 18a is significantly larger than that in Figure 18b, which indicates that our model can receive more abundant location information at the same distance. The lower right part of Figure 18b also has a larger proportion of blanks, indicating that the reinforcement learning with random experience replay has a lower degree of exploration of the environment than our model. Moreover, the distribution of green rings with larger radii in Figure 18b has no obvious directional trend.

#### 4.3.2. Comparison with Brain-Inspired Model

Huang et al.’s [34] brain-inspired model incorporates the olfactory system, which uses odor as a stable potential energy field to ensure the stable propagation of reward signals. However, there are many important resources in nature that do not have odors, such as water, in which case the reward signal cannot be transmitted in the form of smells. In addition, in engineering applications, odor-related sensors are not common in indoor mobile robots, and it is more difficult to use olfactory systems to obtain environmental information than radar systems and vision systems. At the same time, because the reward information is directly transmitted to the agent in the form of a potential field, the agent’s exploration rate of the environment is very low, which this will make the agent abandon its cognition of the environment and go straight to the reward position; then once the reward odor information disappears, the agent will be like a headless fly.

**Figure 19:**
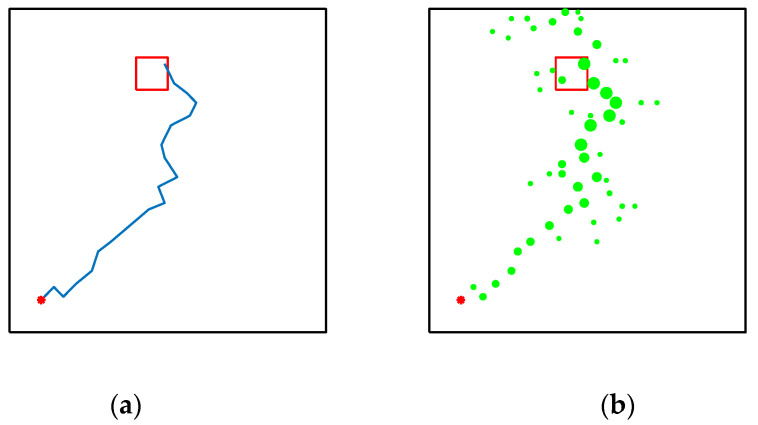

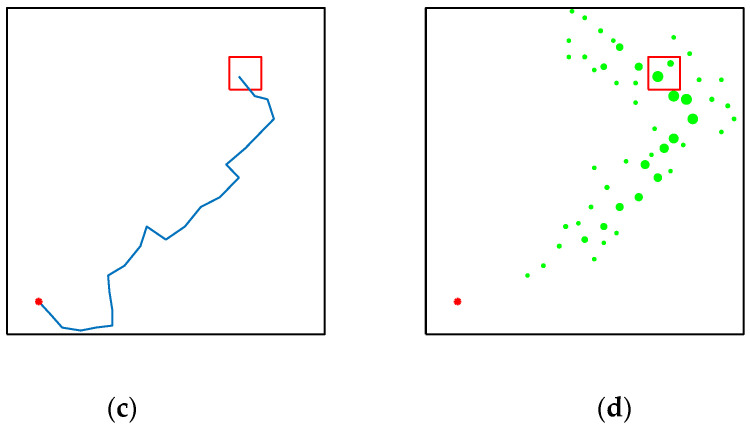
Performance of the brain-inspired model with olfactory system in the water maze experiment. (**a**,**b**) The calculated paths of the model when changing the position of the survival platform. (**c**,**d**) Signal propagation diagrams for navigation at different locations of the survival platform.

**Figure 20:**
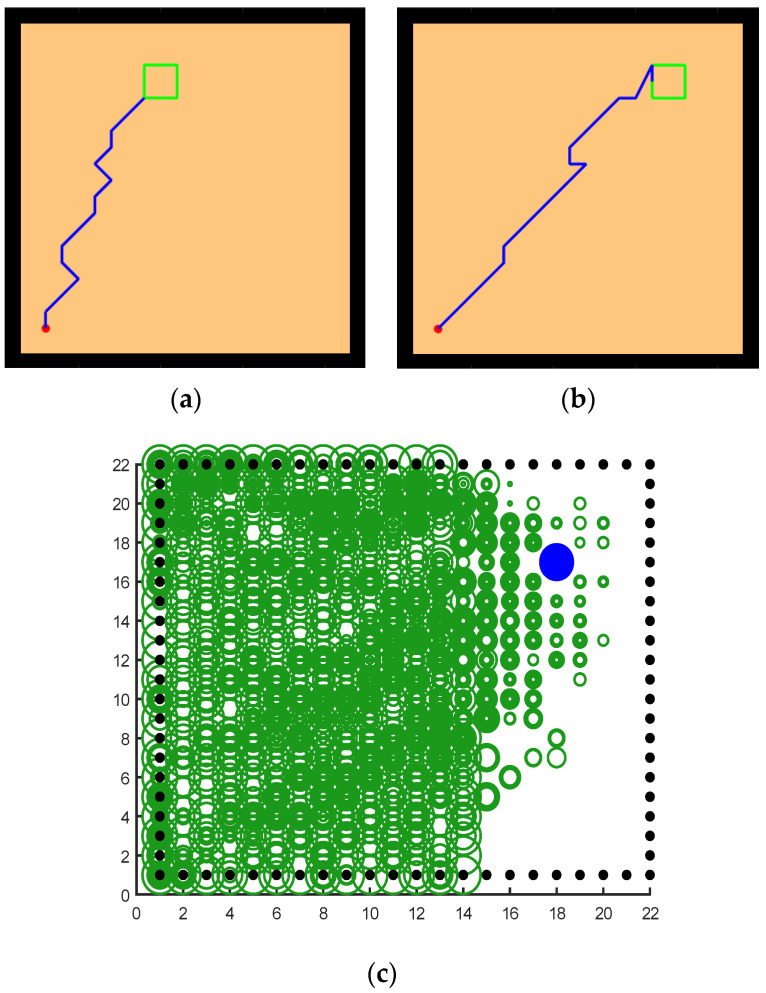
How our model behaves in a water maze experiment. (**a**,**b**) The calculated paths of the model when changing the position of the survival platform. (**c**) The signal propagation diagram for navigation.

Figure 19 shows the performance of their model in the water maze, which starts with a low number of steps due to the lack of environmental exploration, and the reward information is pointed directly to the location of the target. Figure 19b shows that almost all of the environmental signals received by the agent are pointing to the location of the target, and the other spaces in the water maze environment are almost unknown, which is extremely detrimental to the agent’s cognition and learning. As can be seen in Figure 19d, the reward signal is almost out of touch with the agent, which is a very dangerous state. Figure 19c, from the navigation path of the agent, shows that the agent does not have a good grasp of the direction at the beginning of the navigation. On the contrary, although our model needs more environmental exploration and learning in the early stage, this is because our model does not have any hints about the target location information and relies entirely on the ability of environmental cognition and learning to search for rewards. As can be seen in Figure 20c, our model explores the environment much more than the brain-inspired model with olfactory system. In the later stages of exploration, because our model learns and recognizes more about the environment, it can also reach the survival platform along a better path as shown in Figure 20a,b.

Compared with the open field, the signal attenuation problem in complex environments is more serious. Khajeh-Alijani et al. also focused on the problem of neuronal signal attenuation, and they proposed a phase-encoding scheme that can span multiple spatial scales within a single network, and they demonstrated the navigation of complex mazes. Since their research aimed at similar problems, we will compare our model with that of Khajeh-Alijani et al. in navigation experiments in the complex maze they designed to demonstrate the feasibility of our model in complex environments [33].

**Figure 21:**
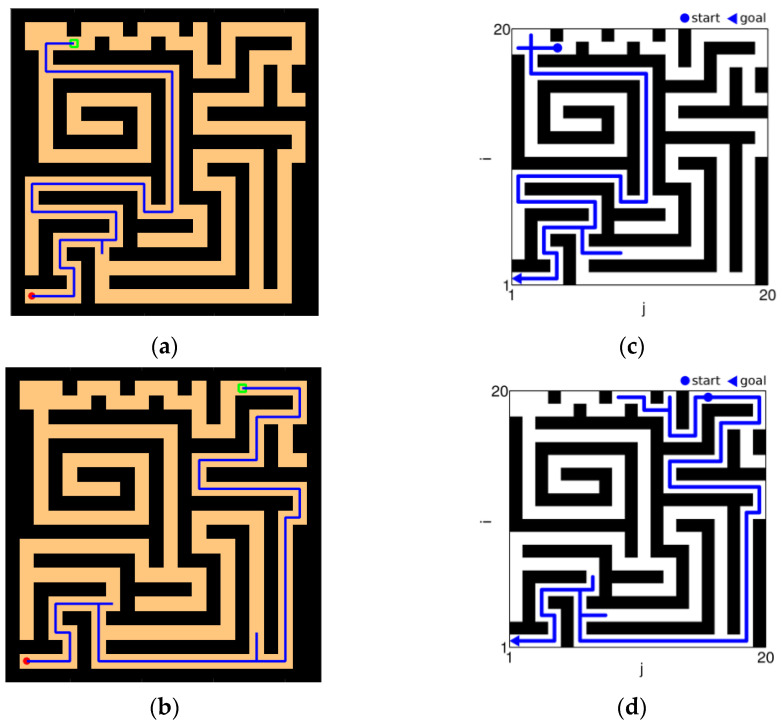
Results of complex maze experiments and comparison of results. (**a**,**b**) The results of our model navigation, where the red circles are the starting points and the green squares are the ending points. (**c**,**d**) The navigation results of the model provided by Khajeh-Azadeh et al. (**a**) Our result in task 1. (**b**) Our result in task 2. (**c**) Contrast result in task 1. (**d**) Contrast result in task 2.

**Figure 22:**
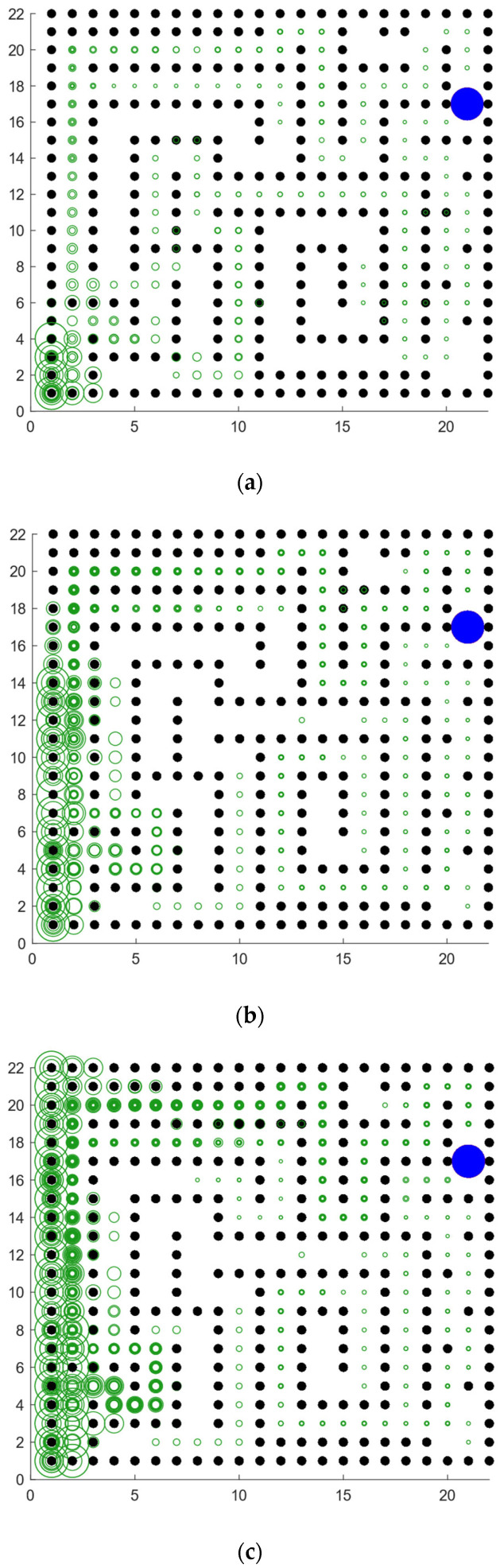
Signal propagation diagrams of our model in a complex maze. The agent starts from the lower left corner of the map (coordinates are (2, 2)) and reaches the blue circle in the upper right corner to get the reward. (**a**) Signal propagation map at the beginning of exploration. (**b**) Signal propagation map for the interim period of exploration. (**c**) Signal propagation map at the later stage of exploration.

As shown in Figure 21, Figure 21a,b are the results of our model navigating in this environment: The red circle represents the starting point, and the green square represents the position of the goal. Figure 21c,d are the complex maze navigation paths provided by Khajeh-Alijani et al. [33]. It can be seen that our model has fewer bifurcation paths in the navigation process and is more robust in complex environments. Figure 22 shows a graph of the signal propagation of our model in a complex maze.

In addition, we moved the complex maze experiment to the Gazebo platform (a 3D robot simulation software that can provide a real physical simulation of the environment and a variety of sensor models). Through a 3D simulation experiment with a physics engine, we verified the feasibility of our model in the physical environment and laid the foundation for the physical realization of the model in the future.

**Figure 23:**
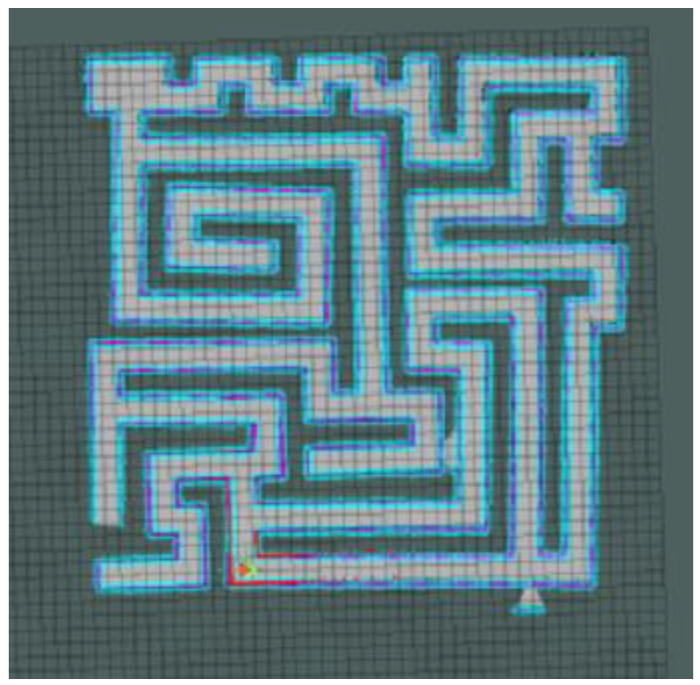
3D maze modeling.

**Figure 24:**
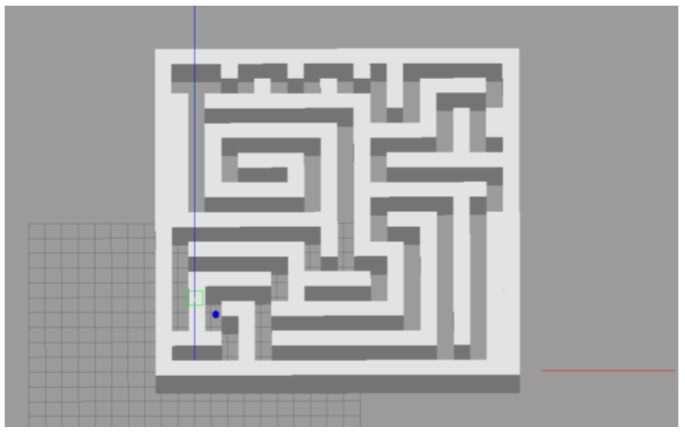
The robot navigates a complex 3D maze.

**Figure 25:**
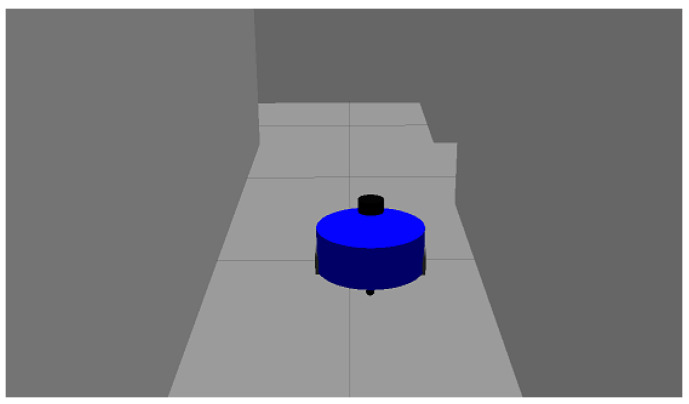
The robot performs the navigation task in the maze.

The robot we used in this experiment was a two-wheel differential robot equipped with lidar. The robot modeling is shown in Figure 9 (in the parameter setting section). The maze was constructed using the Slam Gmapping of the ROS system, as shown in Figure 23 and Figure 24. Finally, our model was mounted on the robot to guide it to complete the navigation task, as shown in Figure 25.

Attached, the running video of the 3D experiment.

## 5. Discussion

The comparative test results show that our model is indeed better than the traditional experience-replay models in terms of exploration rate and speed of finding a better path because our model can simulate a virtual path. The virtual path can provide the agent with more effective samples in a shorter time for its learning, which greatly improves the learning efficiency of the agent.

In general, our Memory-Replay Mechanism first has some similarities with the experience replay in reinforcement learning. Both reuse data to improve the sample efficiency of the reinforcement-learning algorithm. However, the traditional reinforcement learning experience-replay algorithm generally adopts the random experience replay strategy, that is, all the recorded data are randomly sampled and restudied, which gives the reward-independent samples and reward-related samples the same probability of being relearned. Our Memory-Replay Mechanism is inspired by the biological mechanism of hippocampus place cell remapping. Our model simulates the discharge mode of place cells; records the states, actions and position information of the agent; takes the synaptic connection between place cells as the path of information transmission; and uses the reactivation function of the place cells to prevent the information loss caused by signal attenuation in the process of information transmission. In addition, the order of memory replay is associated with reward information [30], and the place cells that are more intimately associated with reward information are preferentially replayed, which further avoids the reuse of reward-irrelevant samples in traditional experience replay and reduces ineffective learning. In this way, the relearning of reward-independent memory is excluded, and the sample utilization and learning efficiency of the model are further improved. At the same time, the memories replayed by our model include not only the real memories in the past but also the virtual paths created through memory integration and reward prediction, none of which the agents have ever really experienced. These characteristics make our model more adaptive, and compared with reinforcement learning algorithms that only refer to past experience, our model has a lower probability of falling into local optimum.

Babichev et al. proposed a computational model of the hippocampus place cell remapping process and mathematically demonstrated that the function of replay helps to learn and maintain the topology of the cognitive map [49]. Our model from a neurophysiological perspective, combined with the place cell-firing model, is modeled at the functional level and proposes a Memory-Replay Mechanism. We have conducted experiments related to signal propagation: As shown in Figure 22a, the agent receives little reward-related information at the starting point, and the trajectory information is complex. Through the further exploration of the environment and the adjustment of the Memory-Replay Mechanism, the agent gradually determines the relatively shorter navigation path. It can be seen from Figure 22b,c that at the adjacent positions of the bifurcation intersection, the green rings are wider, which means there were more cell reactivations in the corresponding position of the agent. This is consistent with findings in neurophysiological studies that animals experienced pauses and increased hippocampus place cell activity at bifurcations [18,28,29]. At the same time, this feature enables our model to have fewer redundant paths during the navigation process, and it can reach the goal location more smoothly with a shorter path.

## 6. Conclusions

Inspired by neurophysiological research, we propose a spatial cognitive model of a Memory-Replay Mechanism that solves the problem of fast signal attenuation in traditional brain-inspired computing model. Different from the experience-replay algorithm used in traditional reinforcement learning, our Memory-Replay Mechanism can integrate past memories and reconstruct the virtual path to improve sample utilization and has preferable biological rationality.

The Morris water maze, a classical spatial cognitive psychology experiment, was simulated to verify the validity of the proposed model. We conducted comparative tests in the Morris water maze and compared our model with Q-learning and random experience-replay models. Our model is superior to the two reinforcement learning models in terms of the length of navigation path, the number of training times to find a better path and the average reward obtained. Moreover, it can be seen from the signal propagation diagram (Figure 15) in the simulated water maze experiment that the signal attenuation amplitude of the model in this paper is smaller than that of the random experience-replay reinforcement learning model, and the agent has a higher degree of exploration of the environment. Experiments were also set up to test the adaptability and robustness of the model in a complex environment. The brain-inspired model research of Khajeh et al. also focuses on the problem of signal attenuation [20]. We tested our model in a complex maze constructed by Khajeh et al. and compared our results with the experimental results of the model by Khajeh et al. The results show that our model has fewer bifurcation paths in a complex environment. At the same time, our model exhibits neuro-physiologically similar results on signal propagation maps. In this paper, the simulation modeling of a 3D environment was also carried out on this basis, which lay the foundation for the subsequent physical experiments.

The model in this paper mainly focuses on the construction of the hippocampus, which simplifies the modeling of the striatum and reduces the influence of other brain regions. Additionally, the sensor used by the agent in this study is lidar, and although it is a common sensor used in the field of robot navigation, vision is the most important perception system for humans and mammals. Most of the research on hippocampus episodic memory is based on visual images, and the remapping of hippocampus place cells is the key to the formation of long-term memory in mammals. Therefore, combining this method with the visual system will be a promising future research direction.

## Figures and Tables

**Table 1:** Parameter settings of the model.

Parameter	Value	Parameter	Value
** α **	0.3	$\sigma_{pc}$	0.7
** ρ **	0.0065	δ	0.5
** γ **	0.9	θ	0.5
** ε **	0.1	$N_{iter}$	1000
** a **	5	p·runs	30
** b **	1	$s_{t}$	0.6
** P **	0.5	$m_{t}$	0.4

**Table 2:** Partial data intercepted within the stabilization interval and their averages.

	Reward Received during Stabilization Period								Average Value
Our model	1	2	3	4	5	6	7	8	7115.15
	7098.053	7116.863	7116.79	7126.873	7142.53	7093.067	7113.307	7127.57	
	9	10	11	12	13	14	15	16	
	7126.763	7115.103	7123.28	7130.467	7104.983	7079.023	7095.157	7132.557	
RL with random experience-replay	1	2	3	4	5	6	7	8	6234.89
	6199.94	6245.663	6258.717	6252.703	6225.827	6183.073	6183.697	6288.38	
	9	10	11	12	13	14	15	16	
	6227.183	6239.723	6241.997	6255.747	6251.603	6236.313	6223.407	6244.197	
Q-learning without experience-replay	1	2	3	4	5	6	7	8	3681.05
	3851	3804.8	3717.9	3563.9	3591.4	3812.5	3736.6	3791.6	
	9	10	11	12	13	14	15	16	
	3788.3	3605.7	3796	3457.2	3803.7	3352.7	3653	3570.5	

## Data Availability

The data are contained within the article.
